# Association of different leisure-time physical activity patterns with risk of sarcopenia among US adults: A cross-sectional study

**DOI:** 10.1097/MD.0000000000050550

**Published:** 2026-09-11

**Authors:** Linbo Peng, Kexin Wang, Mingke You, Bin Shen, Jian Li

**Affiliations:** a Department of Orthopedics and Orthopedic Research Institute, West China Hospital, Sichuan University, Chengdu, China; b Department of Clinical Research Management, Ministry of Science and Technology, West China Hospital, Sichuan University, Chengdu, China; c Sports Medicine Center, West China Hospital, Sichuan University, Chengdu, China.

**Keywords:** leisure-time physical activity, sarcopenia, weekend warrior

## Abstract

Sarcopenia, a progressive loss of skeletal muscle mass and function, poses significant challenges to healthy aging. While leisure-time physical activity (LTPA) has been associated with better muscle-related health, it remains unclear whether condensed activity patterns: such as the “weekend warrior” model: show associations comparable to those of regular activity routines. We analyzed data from 11,780 U.S. adults aged 18 to 60 years in the 2011 to 2018 National Health and Nutrition Examination Survey. Participants were categorized as physically inactive, weekend warriors, or regularly active based on total weekly LTPA volume and frequency. Sarcopenia was defined according to the Foundation for the National Institutes of Health (FNIH) criteria using appendicular lean mass adjusted for body mass index. Logistic regression was used to examine associations, with subgroup analyses based on LTPA frequency, duration, volume, and intensity. Regular LTPA was significantly associated with reduced odds of sarcopenia (odds ratios = 0.654, 95% CI: 0.553–0.773, *P* < .001), whereas the weekend warrior pattern showed a directionally protective but nonsignificant association after full adjustment (odds ratios = 0.684, 95% CI: 0.467–1.001, *P* = .051). Higher frequency, session duration, and vigorous-intensity activity were all associated with lower odds of sarcopenia. Our findings suggest that not only total volume but also the distribution and intensity of LTPA are relevant to sarcopenia risk. These insights may help inform future studies and behavior-based strategies aimed at preserving muscle health in working-age populations.

## 1. Introduction

Over the past 2 centuries, the average life expectancy of humans has experienced a twofold increase in most developed nations. This prolonged longevity has, in turn, contributed to a worldwide escalation in the burden of age-related diseases.^[1]^ Muscle mass and strength begin to decline by approximately 1% per year from 30 years and gradually decline after age 40 and accelerate decline after the age of 50 years.^[2]^ Sarcopenia is defined by the European Working Group on Sarcopenia in Older People 2 as a muscle disease rooted in adverse muscle changes that accrue across the lifetime, with low muscle strength as the key characteristic, low muscle quantity or quality used to confirm the diagnosis, and poor physical performance indicating severe sarcopenia.^[3]^ Sarcopenia is associated with increased risks of falls, frailty, hospitalization, and mortality.^[4]^ It is estimated that the prevalence of sarcopenia ranges from 10% among adults over 65 years and rises to 30% in men over 80 years.^[5,6]^ The progressive demographic shift towards an aging population will likely result in a higher prevalence of sarcopenia and diminished capacity to live independently.^[7]^

Physical activity (PA) plays a vital role in enhancing population health by reducing the risk of cardiovascular disease, hypertension, diabetes, osteoporosis, osteoarthritis, cancer, and all-cause mortality.^[8,9]^ However, the impact of occupational PA on health remains uncertain, with conflicting findings reported in the literature. Some researchers argue that occupational PA might entail prolonged engagement in low-intensity tasks, with inadequate breaks and limited recovery time, which may not confer the same health benefits as leisure-time physical activity (LTPA).^[10,11]^ LTPA typically involves shorter, higher-intensity activity sessions and has been associated with improved cardiovascular health and reduced risk of all-cause, cardiovascular, and cancer mortality.^[12,13]^ According to the 2020 guidelines from the World Health Organization concerning PA and sedentary behaviors for adults, it is recommended to engage in either moderate-intensity aerobic PA for 150 to 300 minutes per week, vigorous-intensity aerobic PA for 75 to 150 minutes per week, or a combination of both intensities that adds up to an equivalent amount. In addition, muscle-strengthening activities involving major muscle groups are recommended on at least 2 days per week to help maintain skeletal muscle mass and function.^[14]^

Regular participation in LTPA has been associated with lower all-cause and cause-specific mortality compared with inactivity.^[15–17]^ Physical inactivity was significantly associated with the risk of possible sarcopenia among middle-aged and older adults from a longitudinal cohort study conducted in China.^[18]^ Previous studies found that LTPA was associated with lower odds for sarcopenia in some small sample cohorts and low- and middle-income countries.^[19–21]^

A comprehensive meta-analysis incorporating data from 7 cohorts showed that both vigorous-intensity and moderate-intensity PA exhibit a comparable reduction in all-cause mortality rates.^[22]^ Engaging in moderate amounts of moderate-to-vigorous physical activity (MVPA) is associated with decreased odds of experiencing probable sarcopenia in older adults.^[23]^ The UK National Health Service recommended distributing MVPA evenly over 4 to 5 days per week or daily.^[24]^

However, it remains unclear whether the same amount of LTPA concentrated within 1 to 2 days per week (“weekend warrior” pattern) shows associations with sarcopenia risk comparable to those observed with more evenly distributed activity (“regularly active” pattern). In addition, it remains unclear whether sarcopenia risk is associated with different dimensions of LTPA, including frequency, session duration, total volume, and intensity. Therefore, we used data from the 2011 to 2018 National Health and Nutrition Examination Survey (NHANES) to investigate the associations of weekend warrior and regularly active LTPA patterns with sarcopenia risk. We also conducted stratified analyses by age, gender, and body mass index (BMI), and further categorized participants according to LTPA frequency, duration, volume, and intensity.

## 2. Methods

### 2.1. Study design and population

The current investigation utilized data from the 2011 to 2018 NHANES conducted by the National Center for Health Statistics. NHANES is a comprehensive cross-sectional population-based survey that combines detailed interviews and thorough physical examinations to assess the vital health and nutritional status of noninstitutionalized individuals within the civilian population of the US.^[25]^ With a commitment to inclusivity and representativeness, NHANES selects approximately 5000 participants annually.

Before the study commenced, all participants provided informed consent, ensuring ethical adherence to research standards, and received approval from the NCHS Ethics Review Board.

**Figure 1.**
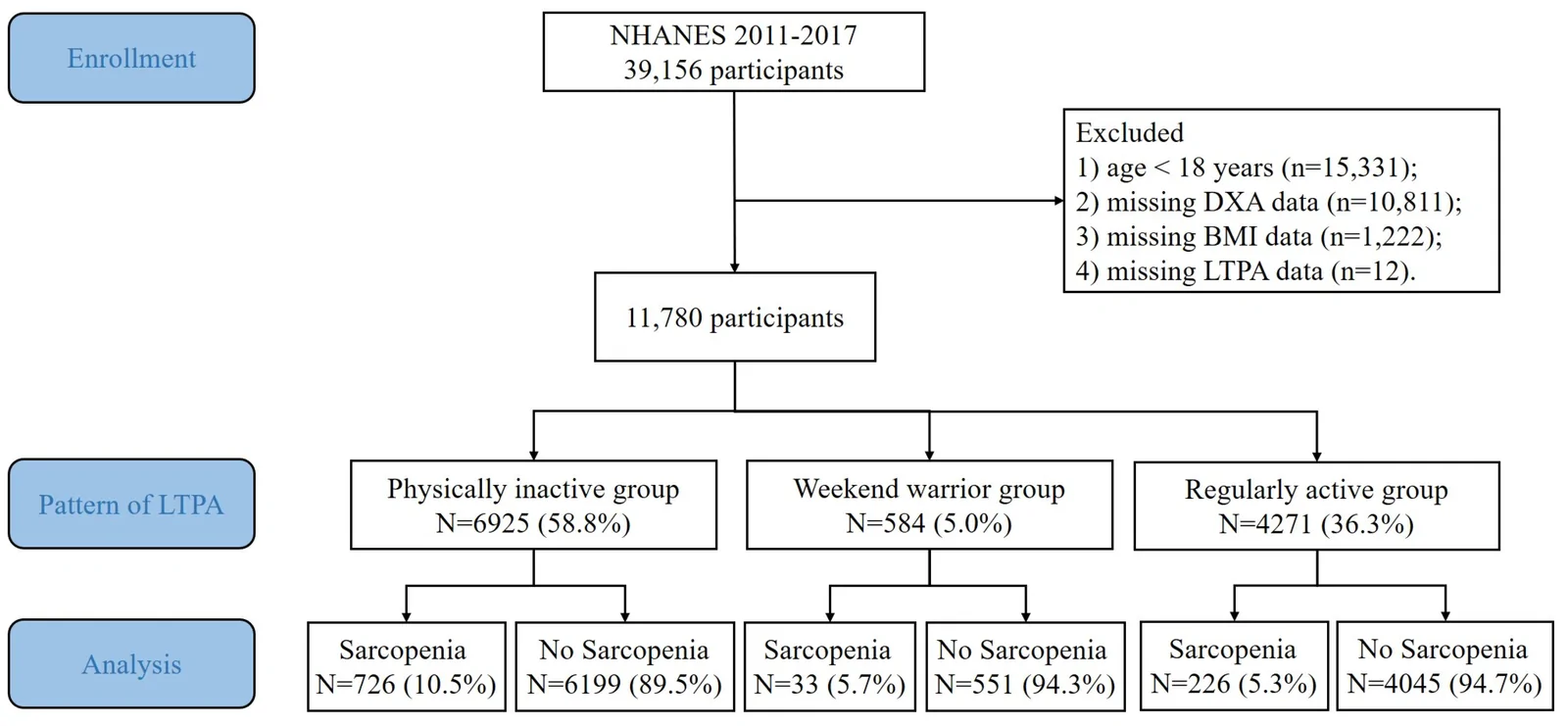
Flow chart of participants for the study process. BMI = body mass index, DXA = Dual-Energy X-ray Absorptiometry, NHANES: National Health and Nutrition Examination Survey, LTPA = leisure-time physical activity.

A total of 39,156 subjects were initially included in the NHANES survey conducted from 2011 to 2018. However, we applied rigorous criteria to select the most relevant data for our investigation. Specifically, we excluded 27,376 participants based on the following criteria: participants under the age of 18 years (n = 15,331); participants without Dual-Energy X-ray Absorptiometry (DXA) data (n = 10,811); participants without BMI data (n = 1222); and participants without LTPA data (n = 12) (shown in Fig. 1). This meticulous exclusion process ensured that our final study dataset comprised 11,780 participants with complete information across all analyzed variables.

### 2.2. Assessment of LTPA

This study assessed the weekly frequency, duration of session, total volume, and intensity of LTPA using a comprehensive set of 6 carefully crafted questions: In a typical week, do you do any vigorous-intensity sports, fitness, or recreational activities that cause large increases in breathing or heart rate like running or basketball for at least 10 minutes continuously? In a typical week, on how many days do you do vigorous-intensity sports, fitness or recreational activities? How much time do you spend doing vigorous-intensity sports, fitness, or recreational activities on a typical day? In a typical week, do you do any moderate-intensity sports, fitness, or recreational activities that cause a small increase in breathing or heart rate, such as brisk walking, bicycling, swimming, or volleyball for at least 10 minutes continuously? In a typical week, on how many days do you do moderate-intensity sports, fitness, or recreational activities? and How much time do you spend doing moderate-intensity sports, fitness, or recreational activities on a typical day?

Moderate physical activity (MPA), vigorous physical activity (VPA), and MVPA were calculated based on the above questions. Total MPA was calculated as the duration per day of moderate-intensity activity multiplied by the weekly frequency of moderate-intensity activity. Total VPA was calculated as the duration per day of vigorous-intensity activity multiplied by the weekly frequency of vigorous-intensity activity and then multiplied by 2. Total MVPA was calculated as total MPA plus total VPA.^[15]^

We classified participants into 3 groups: physically inactive (MVPA < 150 minures/week), weekend warrior (MVPA ≥ 150 minures/week and frequency ≤ 2 sessions/week), and regularly active (MVPA ≥ 150 minutes/week and frequency >2 sessions/week) according to the World Health Organization guidelines for PA. In addition, participants were further classified by frequency (0, 1–2, 3–4, 5–7, and >7 sessions/week), session duration (0, 0–30, 60–120, 120–240, and >240 minutes), total volume (<150, 150–300, 300–450, 450–750, and >750 minutes), and intensity (0, 0–0.5, 0.5, 0.5–1, and 1). Session duration was defined as total MVPA volume divided by frequency. Intensity was defined as the proportion of VPA in MVPA [VPA/(VPA + MPA)] × 100%.

### 2.3. Ascertainment of sarcopenia

Sarcopenia was operationalized according to the Foundation for the FNIH criteria using appendicular lean mass (ALM) adjusted for BMI (ALM/BMI). The diagnostic criterion used in this study involved calculating the ALM-to-BMI ratio.^[26]^ To determine the ALM: BMI ratio, the total lean mass of all 4 limbs was measured using DXA whole body scans obtained from Hologic Discovery model A densitometers (Hologic, Inc., Bedford, Massachusetts). Participants between the ages of 18 and 59 years were eligible for inclusion. Pregnant females were excluded from undergoing DXA examination. Participants excluded from the DXA examination for reasons other than pregnancy were considered eligible nonrespondents. The lean mass was quantified in kilograms. Following the FNIH guidelines, cutoff points for sarcopenia were established at <0.789 kg/m^2^ for men and <0.512 kg/m^2^ for women.^[26]^ Participants with an ALM/BMI ratio below these cutoff values were classified as having sarcopenia.

### 2.4. Covariates

According to prior knowledge, the covariates included were age (classified as 18–44 years and 44–60 years), gender (classified as men and women), BMI (classified as <18.5 kg/m^2^, 18.5–24.9 kg/m^2^, 25.0–29.9 kg/m^2^, and >30 kg/m^2^), and race (classified as Mexican American, other Hispanic, Non-Hispanic white, Non-Hispanic Black, and Other Race), education level (classified as less than high school, high school or equivalent, more than high school, or missing), marital status (married/living with partner, widowed/divorced/separated, never married, or missing), monthly family income (classified as $0-$1649, $1650-$4599, $8400 and over, or missing). BMI was calculated as weight (kg) divided by height squared (m^2^) for body composition assessment. The lifestyle behaviors included alcohol drinks (classified as lifetime abstainer, 0 to 5 oz/day, >5 oz/day, or missing) and cigarette smoking (classified as yes, no, or missing) were considered. In addition, we considered comorbidities (high blood pressure, high cholesterol level, diabetes, weak/failing kidneys, congestive heart failure, coronary heart disease, heart attack, stroke, Chronic Obstructive Pulmonary Disease, liver diseases, asthma, arthritis, gout, cancer or malignancy) into the study.

### 2.5. Statistical analysis

Data were acquired, integrated, and analyzed with appropriate combined weights and strata. Participants were stratified into 3 groups based on their LTPA levels: physically inactive, weekend warrior, and regularly active. Categorical variables were reported as numbers (percentages) and compared using the *χ*2 test. Continuous variables were presented as mean ± SE and compared using one-way ANOVA.

Weighted logistic regression was conducted to examine the adjusted odds ratios (OR) with different covariates in 4 models: model 1 was unadjusted; model 2 adjusted for age, gender, race; model 3 additionally included education level, marital status, monthly family income, and BMI; model 4 additionally included lifestyle behaviors (alcohol drinks, cigarette smoking) and comorbidities (high blood pressure, high cholesterol level, diabetes, weak/failing kidneys, congestive heart failure, coronary heart disease, heart attack, stroke, chronic obstructive pulmonary disease, liver diseases, asthma, arthritis, gout, cancer or malignancy).

To investigate the association between LTPA patterns and sarcopenia in diverse populations, we performed stratification based on age, gender, and BMI. Subgroup analysis was then conducted to explore the potential correlations between LTPA patterns and sarcopenia within each specific subgroup. In addition, we categorized LTPA into different subgroups according to the frequency, duration of session, total volume, and intensity of the activity sessions and examined the adjusted OR. The statistical analyses were conducted using 2-sided tests with a significance level set at *P* < .05. Statistical analysis was performed using R (version 4.0.3) and GraphPad Prism (version 8.3.0).

## 3. Results

### 3.1. The characteristics of participants

The study included a total of 11,780 participants, who were classified into 3 groups based on LTPA: physically inactive group (n = 6925; 58.8%), weekend warrior group (n = 584; 5%), and regularly active group (n = 4271; 36.3%). Comparative analysis revealed a significantly lower proportion of participants with sarcopenia in the weekend warrior and regularly active group than in the physically inactive group (5.7% vs. 5.3% vs. 10.5%, *P* < .001). Table 1 presents the participants’ baseline characteristics according to the LTPA pattern. The proportion of young individuals (18–44 years) within the Weekend Warrior group is significantly greater than that of both the Regularly Active and Physically Inactive groups (76.9% vs. 71.3% vs. 58.0%, *P* < .001). Furthermore, the Weekend Warrior group demonstrates a significantly higher proportion of males compared to the other 2 groups (70.9% vs. 53.3% vs. 45.6%, *P* < .001).

**Table 1:** Baseline characteristics of study participants according to LTPA pattern.

Variable	Pattern of LTPA			Total	*P* value
	Physically inactive	Weekend warrior	Regularly active		
Total, n (%)	6925 (58.8)	584 (5.0)	4271 (36.3)	11780 (100)	
Age, n (%)					<.001
18–44 yr	4018 (58.0)	449 (76.9)	3047 (71.3)	7514 (63.8)	
44–60 yr	2907 (42.0)	135 (23.1)	1224 (28.7)	4266 (36.2)	
Sex, n (%)					<.001
Men	3156 (45.6)	414 (70.9)	2275 (53.3)	5845 (49.6)	
Women	3769 (54.4)	170 (29.1)	1996 (46.7)	5935 (50.4)	
BMI, n (%)					<.001
<18.5 kg/m^2^	170 (2.5)	15 (2.6)	74 (1.7)	259 (2.2)	
18.5–24.9 kg/m^2^	1961 (28.3)	230 (39.4)	1530 (35.8)	3721 (31.6)	
25.0–29.9 kg/m^2^	2061 (29.8)	187 (32.0)	1377 (32.2)	3625 (30.8)	
>30 kg/m^2^	2733 (39.5)	152 (26.0)	1290 (30.2)	4175 (35.4)	
Race, n (%)					<.001
Mexican American	1142 (16.5)	91 (15.6)	573 (13.4)	1806 (15.3)	
Other Hispanic	751 (10.8)	62 (10.6)	417 (9.8%)	1230 (10.4)	
Non-Hispanic White	2278 (32.9)	192 (32.9)	1507 (35.3)	3977 (33.8)	
Non-Hispanic Black	1502 (21.7)	121 (20.7)	916 (21.4)	2539 (21.6)	
Other Race	1252 (18.1)	118 (20.2)	858 (20.1)	2228 (18.9)	
Education level, n (%)					<.001
Less than high school	1479 (21.4)	95 (16.3)	402 (9.4)	1976 (16.8)	
High school or equivalent	1589 (22.9)	128 (21.9)	647 (15.1)	2364 (20.1)	
More than high school	3418 (49.4)	301 (51.5)	2756 (64.5)	6475 (55.0)	
Missing	439 (6.3)	60 (10.3)	466 (10.9)	965 (8.2)	
Marital status, n (%)					<.001
Married/living with partner	3996 (57.7)	276 (47.6)	2185 (51.2)	6459 (54.8)	
Widowed/divorced/separated	1040 (15.0)	61 (10.4)	418 (9.8)	1519 (12.9)	
Never married	1451 (21.0)	185 (31.7)	1202 (28.1)	2838 (24.1)	
Missing	438 (6.3)	60 (10.3)	466 (10.9)	964 (8.2)	
Monthly family income					<.001
$0–$1649	1760 (25.4)	131 (22.4)	828 (19.4)	2719 (23.1)	
$1650–$4599	2518 (36.4)	195 (33.4)	1352 (31.7)	4065 (34.5)	
$8400 and over	1772 (25.6)	182 (31.2)	1541 (36.1)	3495 (29.7)	
Missing	875 (12.6)	76 (13.0)	550 (12.9)	1501 (12.7)	
Alcohol drinks, n (%)					<.001
Lifetime abstainer	1297 (18.7)	81 (13.9)	657 (15.4)	2035 (17.3)	
>0, ≤5 oz/d	3818 (55.1)	362 (62.0)	2781 (65.1)	6961 (59.1)	
>5 oz/d	586 (8.5)	67 (11.5)	317 (7.4)	970 (8.2)	
Missing	1224 (17.7)	74 (12.7)	516 (12.1)	1814 (15.4)	
Cigarette smoking, n (%)					<.001
Yes	2792 (40.3)	227 (38.9)	1284 (30.1)	4303 (36.5)	
No	4022 (58.1)	343 (58.7)	2858 (66.9)	7223 (61.3)	
Missing	111 (1.6)	14 (2.4)	129 (3.0)	254 (2.2)	
High blood pressure	1749 (25.3)	88 (15.1)	741 (17.3)	2578 (21.9)	<.001
High cholesterol level	1714 (24.8)	93 (15.9)	822 (19.2)	2629 (22.3)	<.001
Diabetes	566 (8.2)	26 (4.5)	201 (4.7)	793 (6.7)	<.001
Weak/failing kidneys	140 (2.0)	5 (0.9)	49 (1.1)	194 (1.6)	.001
Congestive heart failure	86 (1.2)	3 (0.5)	23 (0.5)	112 (1.0)	.001
Coronary heart disease	80 (1.2)	2 (0.3)	26 (0.6)	108 (0.9)	.004
Heart attack	113 (1.6)	3 (0.5)	29 (0.7)	145 (1.2)	<.001
Stroke	130 (1.9)	5 (0.9)	26 (0.6)	161 (1.4)	<.001
COPD	115 (1.7)	1 (0.2)	15 (0.4)	131 (1.1)	<.001
Liver diseases	249 (3.6)	8 (1.4)	105 (2.5)	362 (3.1)	<.001
Asthma	1063 (15.4)	109 (18.7)	680 (15.9)	1852 (15.7)	.097
Arthritis	1073 (15.5)	30 (5.1)	389 (9.1)	1492 (12.7)	<.001
Gout	189 (2.7)	10 (1.7)	64 (1.5)	263 (2.2)	<.001
Cancer or malignancy	264 (3.8)	13 (2.2)	133 (3.1)	410 (3.5)	.035
ALM/BMI	0.774 ± 0.194	0.903 ± 0.187	0.855 ± 0.212	0.810 ± 0.205	<.001
Sarcopenia	726 (10.5)	33 (5.7)	226 (5.3)	985 (8.4)	<.001

Categorical variables were reported as case numbers (percentages) and compared using the *χ*2 test. Continuous variables were presented as mean ± SE and compared using one-way ANOVA.

ALM = appendicular lean mass, BMI = body mass index, COPD = chronic obstructive pulmonary disease, LTPA = leisure-time physical activity.

### 3.2. Association of LTPA with sarcopenia

**Figure 2.**
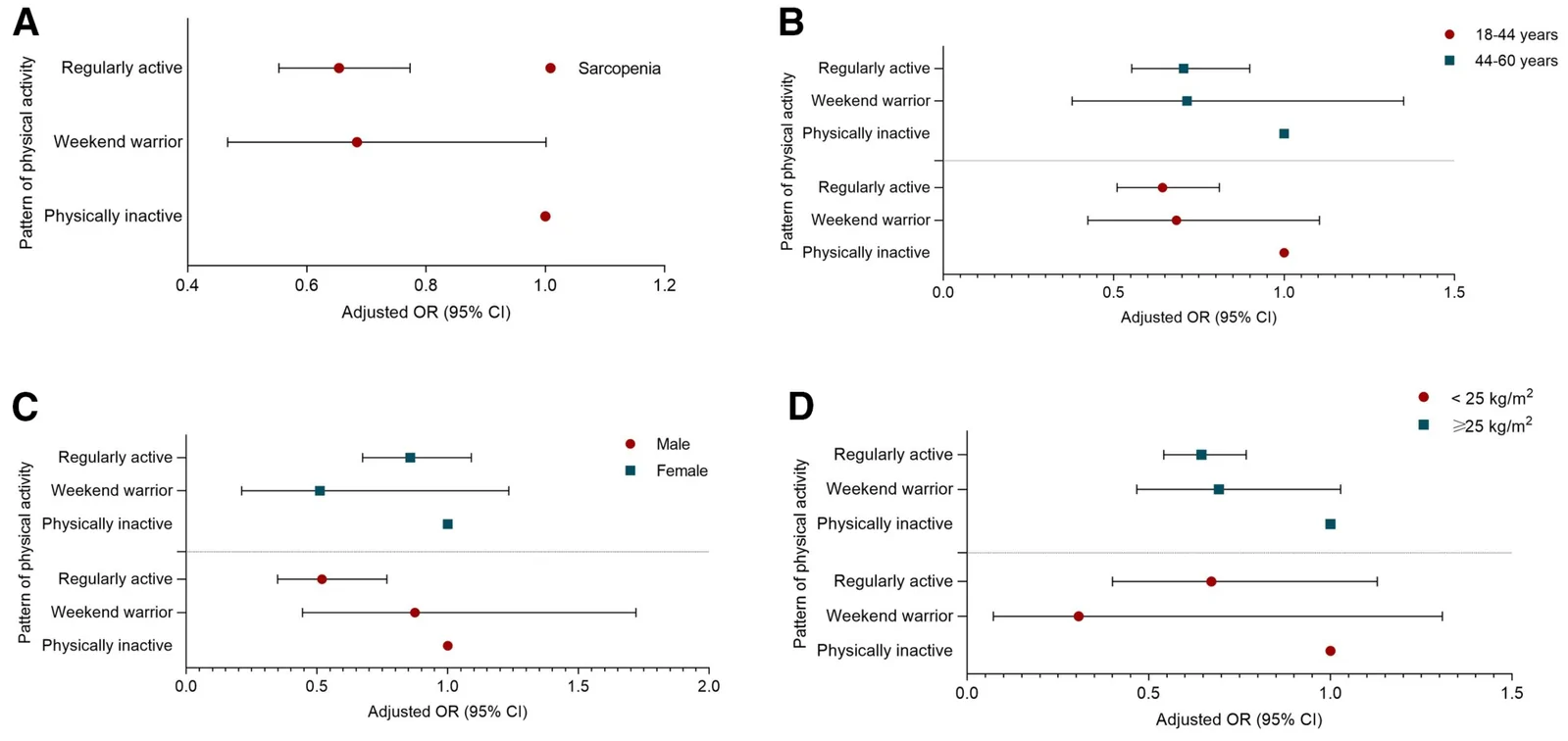
(A) Association of LTPA and sarcopenia estimated by multivariable logistic regression. (B) Association of LTPA and sarcopenia estimated by multivariable logistic regression (stratified by age). (C) Association of LTPA and sarcopenia estimated by multivariable logistic regression (stratified by gender). (D) Association of LTPA and sarcopenia estimated by multivariable logistic regression (stratified by BMI). Using mean values. Bars denote 95% confidence interval. CI = confidence interval, LTPA = leisure-time physical activity, OR = odds ratios.

As shown in Table 2 and Figure 2A, the weekend warrior group showed directionally lower odds of sarcopenia compared with the physically inactive group, although this association did not reach conventional statistical significance after full adjustment (OR = 0.684, 95% CI: 0.467 to 1.001, *P* = .051). However, after adjusting for confounders included in regression model 4, the regularly active group demonstrated significantly lower odds for sarcopenia than the physically inactive group (OR = 0.654, 95% CI: 0.553 to 0.773, *P* < .001).

**Table 2:** Association of LTPA and sarcopenia estimated by multivariable logistic regression.

	Model 1		Model 2		Model 3		Model 4	
	OR (95% CI)	*P* value	OR (95% CI)	*P* value	OR (95% CI)	*P* value	OR (95% CI)	*P* value
All patients								
Physically inactive	Reference	–	Reference	–	Reference	–	Reference	–
Weekend warrior	0.511 (0.357–0.733)	<.001	0.589 (0.408–0.850)	.005	0.687 (0.470–1.006)	.053	0.684 (0.467–1.001)	.051
Regularly active	0.477 (0.409–0.557)	<.001	0.547 (0.466–0.641)	<.001	0.665 (0.563–0.785)	<.001	0.654 (0.553–0.773)	<.001

Adjusted covariates.

model 1: unadjusted.

model 2: Adjusted for age, gender, race.

model 3: model 2 plus education level, marital status, monthly family income, and BMI.

model 4: model 3 plus alcohol drinks, cigarette smoking, high blood pressure, high cholesterol level, diabetes, weak/failing kidneys, congestive heart failure, coronary heart disease, heart attack, Stroke, COPD, liver diseases, asthma, arthritis, gout, cancer or malignancy.

BMI = body mass index, CI = confidence interval, OR = odds ratio.

There were no significant differences in the adjusted odds for sarcopenia between the weekend warrior group and the physically inactive group in both the 18 to 44 years age group and the 44 to 60 years age group. However, upon adjusting for confounding variables in regression model 4, the regularly active group showed significantly lower odds for sarcopenia in both the 18 to 44 years age group (OR = 0.643, 95% CI: 0.510 to 0.810, *P* < .001) and the 44 to 60 years age group (OR = 0.705, 95% CI: 0.553 to 0.899, *P* = .005) (shown in Table 3 and Fig. 2B).

**Table 3:** Association of LTPA and sarcopenia estimated by multivariable logistic regression (stratified by age).

	Model 1		Model 2		Model 3		Model 4	
	OR (95% CI)	*P* value	OR (95% CI)	*P* value	OR (95% CI)	*P* value	OR (95% CI)	*P* value
18–44 yr								
Inactive	Reference	–	Reference	–	Reference	–	Reference	–
Weekend warrior	0.591 (0.376–0.930)	.023	0.548 (0.346–0.870)	.011	0.678 (0.421–1.090)	.109	0.684 (0.424–1.104)	.120
Regularly active	0.498 (0.401–0.618)	<.001	0.510 (0.410–0.636)	<.001	0.628 (0.499–0.789)	<.001	0.643 (0.510–0.810)	<.001
44–60 yr								
Inactive	Reference	–	Reference	–	Reference	–	Reference	
Weekend warrior	0.581 (0.318–1.060)	.077	0.560 (0.303–1.037)	.065	0.681 (0.363–1.279)	.232	0.715 (0.378–1.351)	.301
Regularly active	0.559 (0.446–0.700)	<.001	0.569 (0.452–0.717)	<.001	0.688 (0.541–0.876)	.002	0.705 (0.553–0.899)	.005

Adjusted covariates.

model 1: unadjusted.

model 2: Adjusted for gender, race.

model 3: model 2 plus education level, marital status, monthly family income, and BMI.

model 4: model 3 plus alcohol drinks, cigarette smoking, high blood pressure, high cholesterol level, diabetes, weak/failing kidneys, congestive heart failure, coronary heart disease, heart attack, Stroke, COPD, liver diseases, asthma, arthritis, gout, cancer or malignancy.

BMI = body mass index, CI = confidence interval, OR = odds ratio.

In men, after controlling for confounding variables in regression model 4, the regularly active group showed lower odds of sarcopenia (OR = 0.519, 95% CI: 0.350 to 0.768, *P* = .001). However, in women, neither the weekend warrior group nor the regularly active group showed a significant association with sarcopenia compared to the physically inactive group (shown in Table 4 and Fig. 2C).

**Table 4:** Association of LTPA and sarcopenia estimated by multivariable logistic regression (stratified by gender).

	Model 1		Model 2		Model 3		Model 4	
	OR (95% CI)	*P* value	OR (95% CI)	*P* value	OR (95% CI)	*P* value	OR (95% CI)	*P* value
Male								
Physically inactive	Reference	–	Reference	–	Reference	–	Reference	–
Weekend warrior	0.578 (0.306–1.091)	.091	0.712 (0.373–1.362)	.305	0.812 (0.414–1.590)	.543	0.875 (0.445–1.720)	.698
Regularly active	0.400 (0.276–0.578)	<.001	0.468 (0.321–0.681)	<.001	0.501 (0.339–0.739)	<.001	0.519 (0.350–0.768)	.001
Female								
Physically inactive	Reference	–	Reference	–	Reference	–	Reference	–
Weekend warrior	0.343 (0.151–0.781)	.011	0.387 (0.167–0.896)	.027	0.524 (0.220–1.248)	.144	0.511 (0.212–1.234)	.135
Regularly active	0.574 (0.462–0.713)	<.001	0.655 (0.523–0.820)	<.001	0.836 (0.660–1.058)	.136	0.857 (0.675–1.090)	.208

Adjusted covariates.

model 1: unadjusted.

model 2: Adjusted for age, race.

model 3: model 2 plus education level, marital status, monthly family income, and BMI.

model 4: model 3 plus alcohol drinks, cigarette smoking, high blood pressure, high cholesterol level, diabetes, weak/failing kidneys, congestive heart failure, coronary heart disease, heart attack, Stroke, COPD, liver diseases, asthma, arthritis, gout, cancer or malignancy.

Among the population with BMI ≥ 25 kg/m^2^, the regularly active group demonstrated a significant association with lower odds for sarcopenia (OR = 0.645, 95% CI: 0.541 to 0.768, *P* < .001) after adjusting for confounding variables in regression model 4. However, in the population with BMI < 25 kg/m^2^, neither the weekend warrior group nor the regularly active group showed a significant association with lower odds for sarcopenia compared to the physically inactive group (as shown in Table 5 and Fig. 2D).

**Table 5:** Association of LTPA and sarcopenia estimated by multivariable logistic regression (stratified by BMI).

	Model 1		Model 2		Model 3		Model 4	
	OR (95% CI)	*P* value	OR (95% CI)	*P* value	OR (95% CI)	*P* value	OR (95% CI)	*P* value
<25 kg/m^2^								
Physically inactive	Reference	–	Reference	–	Reference	–	Reference	–
Weekend warrior	0.27 (0.067–1.130)	.073	0.332 (0.080–1.379)	.129	0.289 (0.069–1.214)	.090	0.307 (0.072–1.308)	.110
Regularly active	0.485 (0.300–0.787)	.003	0.565 (0.346–0.924)	.023	0.636 (0.382–1.061)	.083	0.672 (0.400–1.129)	.134
≥25 kg/m^2^								
Physically inactive	Reference	–	Reference	–	Reference	–	Reference	–
Weekend warrior	0.625 (0.429–0.914)	.015	0.668 (0.453–0.986)	.042	0.682 (0.461–1.008)	.055	0.693 (0.467–1.028)	.068
Regularly active	0.512 (0.435–0.604)	<.001	0.579 (0.488–0.686)	<.001	0.630 (0.530–0.749)	<.001	0.645 (0.541–0.768)	<.001

Adjusted covariates.

model 1: unadjusted.

model 2: Adjusted for age, gender, race.

model 3: model 2 plus education level, marital status, and monthly family income.

model 4: model 3 plus alcohol drinks, cigarette smoking, high blood pressure, high cholesterol level, diabetes, weak/failing kidneys, congestive heart failure, coronary heart disease, heart attack, Stroke, COPD, liver diseases, asthma, arthritis, gout, cancer or malignancy.

BMI = body mass index, CI = confidence interval, OR = odds ratio.

### 3.3. Association of LTPA frequency, duration of session, total volume, and intensity with sarcopenia

**Figure 3.**
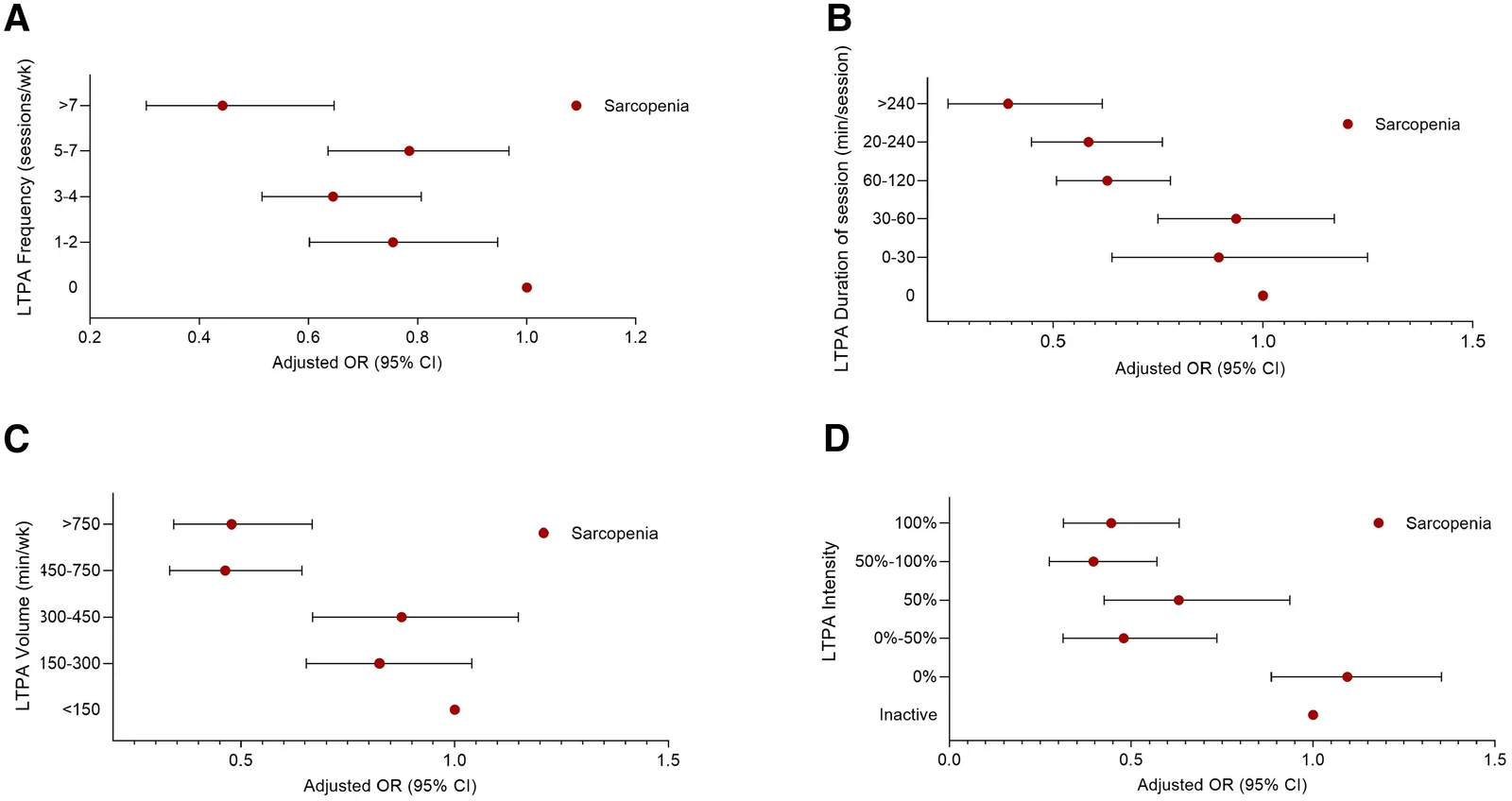
(A) Association of LTPA frequency with sarcopenia estimated by multivariable logistic regression. (B) Association of LTPA duration of session with sarcopenia estimated by multivariable logistic regression. (C) Association of LTPA total volume with sarcopenia estimated by multivariable logistic regression. (D) Association of LTPA intensity with sarcopenia estimated by multivariable logistic regression. CI = confidence interval, LTPA = leisure-time physical activity, OR = odds ratios.

Compared to individuals who do not engage in any LTPA, participants engaging in LTPA 1–2 times per week or more (including 3–4 times per week, 5–7 times per week, and more than 7 times per week) were associated with lower odds of sarcopenia (OR = 0.755, 95% CI: 0.602 to 0.947; OR = 0.645, 95% CI: 0.515 to 0.807; OR = 0.785, 95% CI: 0.636 to 0.968; OR = 0.443, 95% CI: 0.303 to 0.647, respectively) (as shown in Table 6 and Fig. 3A).

**Table 6:** Association of LTPA frequency, duration of session, total volume, and intensity with sarcopenia estimated by multivariable logistic regression.

	Model 1		Model 2		Model 3		Model 4	
	OR (95% CI)	*P* value	OR (95% CI)	*P* value	OR (95% CI)	*P* value	OR (95% CI)	*P* value
Frequency (sessions/wk)								
0	Reference	–	Reference	–	Reference	–	Reference	–
1–2	0.561 (0.455–0.692)	<.001	0.635 (0.512–0.788)	<.001	0.744 (0.595–0.931)	.010	0.755 (0.602–0.947)	.015
3–4	0.472 (0.383–0.581)	<.001	0.545 (0.440–0.674)	<.001	0.637 (0.510–0.796)	<.001	0.645 (0.515–0.807)	<.001
5–7	0.562 (0.463–0.682)	<.001	0.643 (0.527–0.785)	<.001	0.777 (0.631–0.956)	.017	0.785 (0.636–0.968)	.024
>7	0.286 (0.200–0.409)	<.001	0.332 (0.230–0.477)	<.001	0.442 (0.304–0.643)	<.001	0.443 (0.303–0.647)	<.001
Duration of session (min/session)								
0	Reference	–	Reference	–	Reference	–	Reference	–
0–30	0.798 (0.584–1.091)	.157	0.871 (0.632–1.201)	.399	0.885 (0.635–1.234)	.472	0.894 (0.640–1.249)	.510
30–60	0.704 (0.575–0.863)	.001	0.780 (0.632–0.961)	.020	0.954 (0.764–1.189)	.673	0.936 (0.749–1.170)	.562
60–120	0.440 (0.361–0.537)	<.001	0.501 (0.408–0.614)	<.001	0.610 (0.494–0.755)	<.001	0.629 (0.508–0.779)	<.001
120–240	0.403 (0.316–0.515)	<.001	0.459 (0.357–0.590)	<.001	0.581 (0.447–0.754)	<.001	0.584 (0.448–0.760)	<.001
>240	0.252 (0.163–0.389)	<.001	0.323 (0.208–0.503)	<.001	0.379 (0.241–0.596)	<.001	0.392 (0.249–0.617)	<.001
Total volume (min/wk)								
Physically inactive	Reference	–	Reference	–	Reference	–	Reference	–
150–300	0.660 (0.532–0.819)	<.001	0.715 (0.573–0.892)	.003	0.828 (0.658–1.042)	.108	0.824 (0.653–1.040)	.103
300–450	0.592 (0.459–0.764)	<.001	0.677 (0.522–0.879)	.003	0.843 (0.644–1.104)	.214	0.876 (0.667–1.149)	.338
450–750	0.337 (0.247–0.460)	<.001	0.369 (0.269–0.506)	<.001	0.456 (0.330–0.632)	<.001	0.463 (0.333–0.643)	<.001
>750	0.318 (0.233–0.436)	<.001	0.389 (0.282–0.536)	<.001	0.469 (0.337–0.653)	<.001	0.478 (0.342–0.666)	<.001
Intensity (VPA/(VPA + MPA))*100%								
Physically inactive	Reference	–	Reference	–	Reference	–	Reference	–
0%	0.943 (0.775–1.148)	.559	0.985 (0.805–1.206)	.883	1.078 (0.874–1.329)	.484	1.094 (0.885–1.353)	.406
0%–50%	0.351 (0.233–0.527)	<.001	0.397 (0.262–0.600)	<.001	0.475 (0.311–0.725)	.001	0.480 (0.313–0.735)	.001
50%	0.515 (0.356–0.746)	<.001	0.550 (0.377–0.804)	.002	0.608 (0.411–0.900)	.013	0.631 (0.426–0.936)	.022
50%–100%	0.247 (0.174–0.350)	<.001	0.295 (0.207–0.421)	<.001	0.395 (0.274–0.568)	<.001	0.396 (0.275–0.571)	<.001
100%	0.292 (0.209–0.407)	<.001	0.343 (0.245–0.481)	<.001	0.440 (0.311–0.623)	<.001	0.445 (0.314–0.632)	<.001

Adjusted covariates.

model 1: unadjusted.

model 2: Adjusted for age, gender, race.

model 3: model 2 plus education level, marital status, monthly family income, and BMI.

model 4: model 3 plus alcohol drinks, cigarette smoking, high blood pressure, high cholesterol level, diabetes, weak/failing kidneys, congestive heart failure, coronary heart disease, heart attack, Stroke, COPD, liver diseases, asthma, arthritis, gout, cancer or malignancy.

BMI = body mass index, CI = confidence interval, OR = odds ratio.

In comparison to individuals who do not engage in any LTPA, participants with higher duration of activity per session, precisely 60 to 120 minutes/session or more (including 120–240 minutes/session and >240 minutes/session), exhibited lower odds of sarcopenia (OR = 0.629, 95% CI: 0.508 to 0.779; OR: 0.584, 95% CI: 0.448 to 0.760; OR = 0.392, 95% CI: 0.249 to 0.617, respectively). On the other hand, participants with a lower duration of activity per session, including 0 to 30 minutes/session and 30 to 60 minutes/session, did not show a significant association with lower odds for sarcopenia when compared to individuals with 0 minutes/session of LTPA (as shown in Table 6 and Fig. 3B).

When compared to physically inactive individuals, participants engaging in a total volume of 450 to 750 minutes/week or more than 750 minutes/week exhibited lower odds of sarcopenia (OR = 0.463, 95% CI: 0.333 to 0.643; OR = 0.478, 95% CI: 0.342 to 0.666, respectively). However, no significant association was found between participants with a total volume of 150 to 300 minutes/week or 300 to 450 minutes/week and lower odds for sarcopenia compared to the physically inactive group (as shown in Table 6 and Fig. 3C).

Regarding the intensity, participants with an intensity of 0% to 50% VPA or more (50% VPA, 50%–100% VPA, 100% VPA) demonstrated lower odds of sarcopenia compared to physically inactive individuals (OR = 0.480, 95% CI: 0.313 to 0.735; OR = 0.631, 95% CI: 0.426 to 0.936; OR = 0.396, 95% CI: 0.275 to 0.571, OR = 0.445, 95% CI: 0.314 to 0.632, respectively). However, participants with an intensity of 0% VPA did not show a significant association with lower odds for sarcopenia compared to the physically inactive group (as shown in Table 6 and Fig. 3D).

## 4. Discussion

This study examined the association of different LTPA patterns with sarcopenia risk in a large cohort of 11,780 adults in the US. To our knowledge, this is among the first population-based studies to examine how specific LTPA behavioral patterns: including the weekend warrior model: relate to sarcopenia risk across multiple dimensions of frequency, duration, volume, and intensity.

The results showed that the prevalence of sarcopenia was 8.4% in US adults aged 18 to 60 years. After adjusting for potential confounders, regularly active participants demonstrated lower odds of sarcopenia compared to physically inactive individuals. The weekend warrior pattern showed directionally lower odds of sarcopenia after full adjustment, although this association did not reach conventional statistical significance. This finding may suggest a potential protective trend that warrants further investigation.

In addition, our study demonstrated that regularly active participants exhibited lower odds of sarcopenia compared to physically inactive individuals across various subgroups, including the 18 to 44 years and 44 to 60 years age groups, male population, and individuals with BMI ≥ 25 kg/m^2^, when the data were stratified by age, gender, and BMI.

Furthermore, participants engaging in at least LTPA 1 to 2 times each week or spending 60 to 120 minutes per session had lower odds of sarcopenia compared to completely inactive participants (those with a frequency or duration of LTPA sessions equal to 0). Participants who engaged in a total volume of more than 450 minutes of LTPA each week or included VPA within their LTPA demonstrated lower odds of sarcopenia when compared to physically inactive participants.

Our findings, indicating that LTPA was associated with lower odds of sarcopenia, are consistent with several previous studies in the field.^[19–21]^ In older adults with obesity and metabolic syndrome, increased LTPA is significantly associated with favorable health outcomes. Specifically, higher LTPA levels are linked to a reduction in fat mass and a concurrent improvement in bone mass and lower-limb muscle strength.^[21]^ Among older adults from low- and middle-income countries, a cross-sectional study revealed a significant association between insufficient LTPA and the prevalence of sarcopenia.^[19]^ In a small cohort study of nursing home older residents, it was observed that individuals engaging in LTPA for 1 hour or more per day had a reduced likelihood of presenting with sarcopenia.^[20]^ Compared with these previous studies, our study included a large sample of adults from a nationally representative US population. Previous studies with the largest sample sizes were primarily conducted in low- and middle-income countries, which may have limitations in terms of generalizability.^[19]^ Furthermore, our study is among the first to examine the association between detailed LTPA patterns and sarcopenia risk in a large US adult population. These distinctive aspects of our study contribute valuable insights to the existing literature and provide a more comprehensive understanding of the association between LTPA pattern and sarcopenia risk in diverse populations.

Our findings indicate that the weekend warrior pattern showed a directionally protective association with sarcopenia risk, although it did not reach conventional statistical significance after full adjustment. It has been found that the weekend warrior pattern is associated with similarly lower odds of cardiovascular outcomes and all-cause mortality rates compared to the regularly active population.^[15,24]^ More recently, several studies have directly examined the association between weekend warrior PA patterns and sarcopenia-related outcomes. Wang et al reported that both weekend warrior and regularly active MVPA patterns were associated with lower odds of sarcopenia among older Chinese adults, based on the 2019 AWGS criteria.^[27]^ Similarly, Kim et al found that the weekend warrior pattern was associated with a lower prevalence of sarcopenia than both inactivity and regular activity in middle-aged and older adults, possibly because weekend warriors accumulated a greater amount of vigorous-intensity activity.^[28]^ In addition, Liu et al used accelerometer-derived PA data and showed that concentrating MVPA within 1–2 days per week was associated with lower odds of incident probable sarcopenia, confirmed sarcopenia, and falls over long-term follow-up.^[29]^

The different associations observed between the weekend warrior pattern and regular PA may partly reflect the specific nature of sarcopenia-related outcomes. Concentrating LTPA within 1 to 2 days may not be associated with the same magnitude of lower sarcopenia odds as more regularly distributed activity in all populations. However, current guidelines recommend muscle-strengthening activities on at least 2 days per week, which may be compatible with a weekend warrior pattern when sufficient resistance-based or vigorous-intensity activity is accumulated. Therefore, the weekend warrior pattern should not be interpreted as inherently inconsistent with muscle-strengthening recommendations. Further prospective studies are needed to clarify how activity distribution, intensity, and exercise type are associated with sarcopenia-related outcomes.^[30]^ These recent findings differ somewhat from our results, in which the weekend warrior pattern showed only a directionally protective trend after full adjustment.^[27–29]^ These discrepancies may reflect differences in sarcopenia definitions, PA assessment methods, age structure, and vigorous-intensity activity composition. Unlike these recent studies, our analysis used DXA-derived ALM/BMI and self-reported NHANES data in US adults aged 18 to 60 years.

In our study, regularly active participation was associated with lower odds of sarcopenia in males compared with physically inactive individuals, whereas this association was not evident among females. This finding differs from Jacob et al who reported a stronger association between low LTPA and sarcopenia in women but not in men.^[19]^ These inconsistencies may be partly explained by differences in health status, age distribution, occupational activity, dietary patterns, and baseline characteristics across cohorts.^[31]^

Our study found that regular LTPA was associated with lower odds of sarcopenia among participants with BMI ≥ 25 kg/m^2^ compared with physically inactive individuals. Similarly, Rosique-Esteban et al reported inverse associations between sarcopenia and LTPA in older adults with obesity and metabolic syndrome.^[21]^ In our study, among participants with BMI below 25 kg/m^2^, no association was found between low LTPA and an increased risk of sarcopenia. The relatively low prevalence of sarcopenia in this subgroup may have contributed to these results. Prior studies have reported an inverse relationship between higher BMI and sarcopenia prevalence in adults over the age of 60.^[32,33]^ Nevertheless, in our cohort, adults under 60 years with BMI >25 kg/m^2^ had an elevated risk of sarcopenia. These findings suggest that the association between LTPA and lower sarcopenia risk may be more apparent among individuals with higher BMI than among those with BMI below 25 kg/m^2^.

Our findings indicate that individuals who engaged in LTPA more than 1 to 2 times per week or for 60 to 120 minutes or more per session had lower odds of sarcopenia compared with those who did not engage in any LTPA. Additionally, participants with a total LTPA volume exceeding 450 to 750 minutes per week or those incorporating VPA within their LTPA also demonstrated lower odds of sarcopenia than physically inactive individuals. These findings suggest that even relatively low frequency or duration of LTPA may be associated with reduced sarcopenia risk. Similarly, Zhao et al reported associations between PA intensity, frequency, duration, and weekly volume and the risk of possible sarcopenia in middle-aged and older adults.^[18]^ In contrast, Zhao et al defined possible sarcopenia using handgrip strength and the 5-time chair stand test, whereas our study utilized DXA-derived ALM/BMI to assess sarcopenia. In addition, we focused specifically on LTPA, which is more standardized and widely used in epidemiological research compared with general PA measures.^[10]^ As a result, our findings may have greater comparability and generalizability. Our study defined intensity based on the proportion of VPA within LTPA, consistent with previous literature.^[15]^ LTPA with only MPA participation was not significantly associated with reduced sarcopenia risk, whereas inclusion of VPA was associated with lower risk. Notably, our study further highlights the potential advantage of incorporating VPA within LTPA. Previous studies have generally indicated that MVPA is associated with reduced sarcopenia risk.^[19–21,34]^

## 5. Limitations

Several limitations in this study should be acknowledged. First, due to the design of the cross-sectional study, we could not draw definitive causal conclusions from the correlations we observed. Hence, we could not examine the temporal relationship between LTPA and the onset of sarcopenia. Second, considering the study’s observational framework, residual confounding may still exist, despite our efforts to control for numerous potential confounding variables. Third, our research is confined to the adult population ranging from 18 to 60 years old. Consequently, the findings may not be directly applicable to adults over the age of 60, a demographic group that merits greater focus on sarcopenia. Fourth, our assessment of LTPA relied on participants’ self-reporting, a factor that could potentially introduce recall bias into our findings. Finally, the NHANES dataset does not provide detailed information on specific types of PA (e.g., aerobic exercise, resistance training, or other forms of activity). This distinction is important, as different types of PA may have varying effects on skeletal muscle mass and function, with resistance training being particularly relevant for sarcopenia prevention. Future studies using device-based PA assessment and more detailed information on exercise type may help clarify whether specific activity patterns, particularly vigorous-intensity or resistance-based weekend warrior activity, are associated with lower odds of sarcopenia.

## 6. Conclusions

Regular engagement in LTPA was significantly associated with reduced odds of sarcopenia compared with physical inactivity. The weekend warrior pattern showed a directionally protective association, although it did not reach conventional statistical significance after full adjustment. In addition, higher frequency, longer duration, greater total volume, and engagement in vigorous-intensity activity were associated with lower odds of sarcopenia. These findings highlight the relevance of total activity volume, distribution, and intensity of LTPA in relation to sarcopenia risk and suggest that the potential association between weekend warrior activity and sarcopenia-related outcomes warrants further investigation.

## Acknowledgments

This paper uses data from National Health and Nutrition Examination Surveys (NHANES) conducted by the National Center for Health Statistics (NCHS). Before the study commenced, all participants provided informed consent, ensuring ethical adherence to research standards, and received approval from the NCHS Ethics Review Board. Finally, the authors of this manuscript certify that they comply with the ethical guidelines for authorship and publishing in the Medicine.

## Author contributions

**Conceptualization:** Linbo Peng, Bin Shen, Jian Li.

**Data curation:** Linbo Peng, Kexin Wang, Jian Li.

**Formal analysis:** Linbo Peng, Mingke You.

**Funding acquisition:** Linbo Peng, Bin Shen.

**Investigation:** Linbo Peng, Kexin Wang.

**Methodology:** Linbo Peng, Kexin Wang, Mingke You.

**Software:** Linbo Peng, Mingke You.

**Visualization:** Linbo Peng, Kexin Wang, Mingke You.

**Project administration:** Kexin Wang.

**Supervision:** Kexin Wang, Bin Shen, Jian Li.

**Validation:** Mingke You, Jian Li.

**Resources:** Bin Shen, Jian Li.

**Writing – original draft:** Linbo Peng, Kexin Wang.

**Writing – review & editing:** Bin Shen, Jian Li.
